# Editorial: Urinary lithiasis in children

**DOI:** 10.3389/fped.2025.1682402

**Published:** 2025-09-02

**Authors:** Alberto Parente, Rubén Ortiz, Sonia Pérez-Bertólez, Francisco Javier Reed

**Affiliations:** ^1^ Reina Sofia University Hospital, Cordoba, Spain; ^2^ Hospital Materno Infantil Gregorio Maranon, Madrid, Spain; ^3^ Hospital Sant Joan de Deu, Barcelona, Spain; ^4^ Hospital Dr Exequiel Gonzalez Cortes, San Miguel, Chile

**Keywords:** children, lithiasis in children, urinary lithiasis, endourology, stones

**Editorial on the Research Topic**
Urinary lithiasis in children

The selected articles collectively contribute valuable knowledge that enhances the understanding, prevention, and treatment of pediatric urolithiasis—a growing health concern worldwide. While only one of the six articles directly focuses on kidney stones in children, the others offer crucial insights into associated urinary tract conditions, surgical innovations, and risk factors that are highly relevant to comprehensive stone management.

First, studies such as the one on global trends in pediatric urolithiasis (Zhang et al.) provide a macro-level epidemiological perspective, identifying high-risk regions and age groups. This data-driven approach supports targeted public health interventions and resource allocation, especially in countries facing rising prevalence.

This large-scale study leveraged Global Burden of Disease (GBD) (1) data to examine pediatric urolithiasis prevalence in Brazil, Russia, India, China, and South Africa (BRICS). While global case numbers have raised, the age-standardized prevalence rate (ASPR) declined slightly. India exhibited the highest prevalence, while Russia led in ASPR. Notably, South Africa showed insufficient early prevention, especially in the 0–4 age group, and Brazil demonstrated the fastest worsening trend. Conversely, China showed consistent improvement. Subgroup analyses emphasized rising rates in the 10–14 age group. The study emphasizes targeted prevention strategies and resource allocation tailored to national contexts.

Second, the case-report using a tip-flexible vacuum-assisted ureteral access sheath (Cheng et al.) represents a major advancement in surgical technique, offering a safer and more effective method for removing stones post-pyeloplasty. This is critical in preventing recurrence and preserving renal function, especially in children with structural anomalies like UPJO (2). This case report described the successful use of a tip-flexible vacuum-assisted ureteral access sheath (FV-UAS) in a 13-year-old boy who developed renal stones following pyeloplasty for ureteropelvic junction obstruction (UPJO). The flexible sheath enabled safe navigation and suction-assisted clearance of stones during flexible ureteroscopic laser lithotripsy. Notably, the anastomosis site remained intact, and there were no complications. This novel approach demonstrates high safety and efficacy for post-pyeloplasty lithiasis and is a promising addition to pediatric endourology, offering improved stone-free rates with minimal trauma.

Additionally, procedures described for removing ureteral stents in infants (Zeng et al.) and performing laparoscopic ureteral reimplantation (Shang et al.) highlight minimally invasive solutions that reduce complications and recovery times—factors essential when managing complex urolithiasis cases, which often involve concurrent conditions like hydronephrosis or obstructive uropathy.

Zeng et al. introduced a novel, minimally invasive technique for removing double-J ureteral stents in infants using Prolene sutures under cystoscopy. In 15 infants post-pyeloplasty, the average operative time was just 3.5 min, and no complications occurred, such as urethral or bladder injuries. This method proves particularly valuable in neonates and small infants where traditional grasping forceps are unfeasible due to anatomical constraints. The authors highlight this cost-effective and safe alternative as especially useful in resource-limited or pediatric-specialized settings (3).

Shang et al., evaluated Transabdominal Laparoscopic Ureteral Reimplantation (TALUR) against the traditional Politano-Leadbetter approach in children with primary obstructive megaureter (POM). Results from 41 patients demonstrated that TALUR significantly reduced operative time (76 vs. 96 min), hospital stay, and postoperative ureteral diameter, while maintaining similar rates of complication and surgical success. Follow-up imaging showed hydronephrosis improvement and ureteral patency. TALUR appears to be a viable, minimally invasive alternative with better recovery outcomes, especially when performed at the bladder dome for enhanced exposure.

Moreover, the investigation into excessive sugar intake and overactive bladder (OAB) (Cui et al.) reveals lifestyle factors that may indirectly increase the risk of stone formation through altered voiding patterns, dehydration, or metabolic changes. Children consuming ≥50 g of sugar daily, particularly fructose from fruits and juices, had higher OAB symptom scores and longer symptom duration. These findings underscore the importance of dietary assessment in pediatric urology, suggesting that sugar reduction should be a key component of OAB management strategies. This underscores the need for dietary and behavioral interventions alongside clinical treatments.

Finally, the study on vesicoureteral reflux and renal function deterioration (Yan et al.) reinforces the role of early imaging and diagnosis in preventing long-term renal damage—a complication commonly associated with recurrent stones and infections in pediatric patients. This retrospective study explored the impact of high-grade vesicoureteral reflux (VUR) and intrarenal reflux (IRR) on kidney morphology and function using contrast-enhanced voiding urosonography (ceVUS) in 110 children. Findings revealed that IRR was only observed in VUR grade II or higher, with a strong correlation between IRR severity and higher VUR grades. Patients with high-grade VUR and IRR had significantly reduced kidney size and lower DMSA-based renal function compared to those without reflux. The study supports ceVUS as a radiation-free, sensitive tool for early identification of high-risk children and urges early intervention to prevent long-term renal damage (4).

Together, these studies advocate for an integrated, multidisciplinary approach to pediatric urolithiasis. They emphasize the importance of combining early diagnosis, innovative surgical methods, lifestyle modification, and long-term follow-up to reduce disease burden and improve patient outcomes in children suffering from or at risk of urinary stone disease.
